# Astroviruses in terrestrial Malagasy mammals

**DOI:** 10.1371/journal.pntd.0012263

**Published:** 2024-06-14

**Authors:** Victoria Carcauzon, James P. Herrera, Kayla Kaufman, Fiona Baudino, Natalie Wickenkamp, Toky M. Randriamoria, Voahangy Soarimalala, Steven M. Goodman, Charles L. Nunn, Camille Lebarbenchon, Pablo Tortosa

**Affiliations:** 1 Université de La Réunion, Unité Mixte de Recherche Processus Infectieux en Milieu Insulaire Tropical (UMR PIMIT), CNRS 9192, INSERM 1187, IRD 249, Plateforme Technologique CYROI, Sainte Clotilde, La Réunion, France; 2 Evolutionary Anthropology, Duke University, Durham, North Carolina, United States of America; 3 Duke Lemur Center SAVA Conservation, Durham, North Carolina, United States of America; 4 University of California Santa Barbara, Department of Ecology, Evolution, and Marine Biology, University of California, Santa Barbara, California, United States of America; 5 Association Vahatra, Antananarivo, Madagascar; 6 Field Museum of Natural History, Chicago, Illinois, United States of America; 7 Duke Global Health Institute, Durham, North Carolina, United States of America; Faculty of Science, Ain Shams University (ASU), EGYPT

## Abstract

Small terrestrial mammals are major hosts of infectious agents responsible for zoonotic diseases. Astroviruses (AstVs)–the cause of non-bacterial gastroenteritis mainly affecting young children–have been detected in a wide array of mammalian and avian host species. However, understanding the factors that influence AstV infection within and across hosts is limited. Here, we investigated the impact of land use changes on AstVs in terrestrial small mammals in rural northeastern Madagascar. We sampled 515 small mammals, representing seven endemic and four introduced species. Twenty-two positive samples were identified, all but one of which were found in the introduced species *Mus musculus* and *Rattus rattus* (family Muridae), with a positivity rate of 7.7% (6/78) and 5.6% (15/266), respectively. The non-introduced rodent case was from an endemic shrew-tenrec (family Tenrecidae). We found the highest positivity rate of AstVs infection in brushy regrowth (17.5%, 7/40) as compared to flooded rice fields (4.60%, 8/174), secondary forest (4.1%, 3/74), agroforest (3.6%, 1/28), village (2.61%, 3/115), and semi-intact forest (0%, 0/84). A phylogenetic analysis revealed an association between AstVs and their rodent host species. None of the viruses were phylogenetically related to AstVs previously described in Malagasy bats. This study supports AstV circulation in synanthropic animals in agricultural habitats of Madagascar and highlights the need to assess the spillover risk to human populations in rural areas.

## Author summary

This article describes the diversity of Astroviruses (AstVs) shed by Malagasy terrestrial small mammals and explores the impact of habitat degradation on positivity rate in animal reservoirs. Using a sample of 515 terrestrial small mammals from northeastern Madagascar, we show that AstV infection is almost exclusively restricted to rats and mice, suggesting that AstVs from terrestrial mammals were introduced to Madagascar. A phylogenetic analysis including AstVs sequences from Malagasy small mammals reveals a strong structuring of AstV sequences within host species, indicating limited host switching of these viruses among small mammals. Finally, positivity rates show that some habitat types are particularly conducive to AstV infection in reservoirs, such as rice fields where mice exhibit maximal infection. Altogether, presented data encourage the need for assessing the actual exposure of the local human population to the AstVs in small mammals, especially rice-field farmers.

## Introduction

Astroviruses (AstVs) are single-stranded non-enveloped RNA viruses belonging to the family *Astroviridae*. They are responsible worldwide for 2–8% of non-bacterial gastroenteritis affecting children under five years of age [1], making them the third most common viral agent of acute diarrhea after rota- and noro-viruses [2–4]. AstVs are mainly transmitted via the fecal-oral route or indirectly through consumption of contaminated food or water. Because these viruses can persist in contaminated environments for long periods, non-contact transmission is an important mechanism of spread [5]. Symptoms of AstV-caused gastroenteritis include diarrhea, which can lead to severe disease in immunocompromised children or adults [6]. Recently, other types of AstVs (e.g. HAstV-MLB and HAstV-VA/HMO) have been identified in humans and are thought to be associated with central nervous system infections [7].

AstVs have been detected in more than 80 non-human host species including a wide diversity of small terrestrial mammals (e.g., rodents and shrews) with a nearly global distribution that includes North America [8], Asia [9,10], and Africa [11]. AstVs are also common in bats [12], and have been recently detected in a range of species in the western Indian Ocean, specifically Mozambique [13], Madagascar [14], and Reunion Island [15].

Although no information is available regarding AstV in human populations from Madagascar, enteric diseases are highly prevalent in the country, and these viruses might be of medical concern. Since small mammals, and especially introduced rodents, are commensal to human populations, we investigated AstV circulation in introduced and endemic small terrestrial mammals in a humid forest area of northeastern Madagascar, focusing on viral positivity rate, diversity, and effect of land use and season on viral infection. The study was conducted within and adjacent to Marojejy National Park over three years (2017–2019). The protected area has an exceptional level of vertebrate diversity [16] and the surrounding areas are characterized by different forms of agricultural use. These landscapes are used by local people for a variety of economic and subsistence activities, including rice, vanilla and other agricultural products, harvesting of trees for cooking fuel and house construction, and limited livestock pasture land [17]. We sampled animals in plots along a land use gradient encompassing villages, agricultural zones, secondary forest fragments, and disturbed native forest within the national park.

We hypothesized that habitat disturbance influences AstV transmission in terrestrial small mammals by affecting the community structure of mammal reservoirs and by altering infection positivity rates in reservoirs, such as rice farming activities that may increase viral positivity rate due to non-contact transmission in humid environments. A previous study on bats found an effect of season but no effect of land use on AstV infection prevalence in bats [18]. However, terrestrial small mammals have more limited dispersal capacities than bats, and are in permanent and direct contact with the ground. As a result, it is likely that spatio-temporal dynamics of AstVs shed by bats and terrestrial small mammals are distinct.

## Methods

### Ethics statement

The protocols of animal capture and handling, and collection of biological material were approved by the Institutional Animal Care and Use Committee of Duke University (protocol numbers A002-17-01 & A262-19-12) and by Malagasy authorities (permit numbers 289/17 & 146/18—MEEF/SG/DGF/DSAP/SCB).

### Study sites and sampling seasons

**Fig 1:**
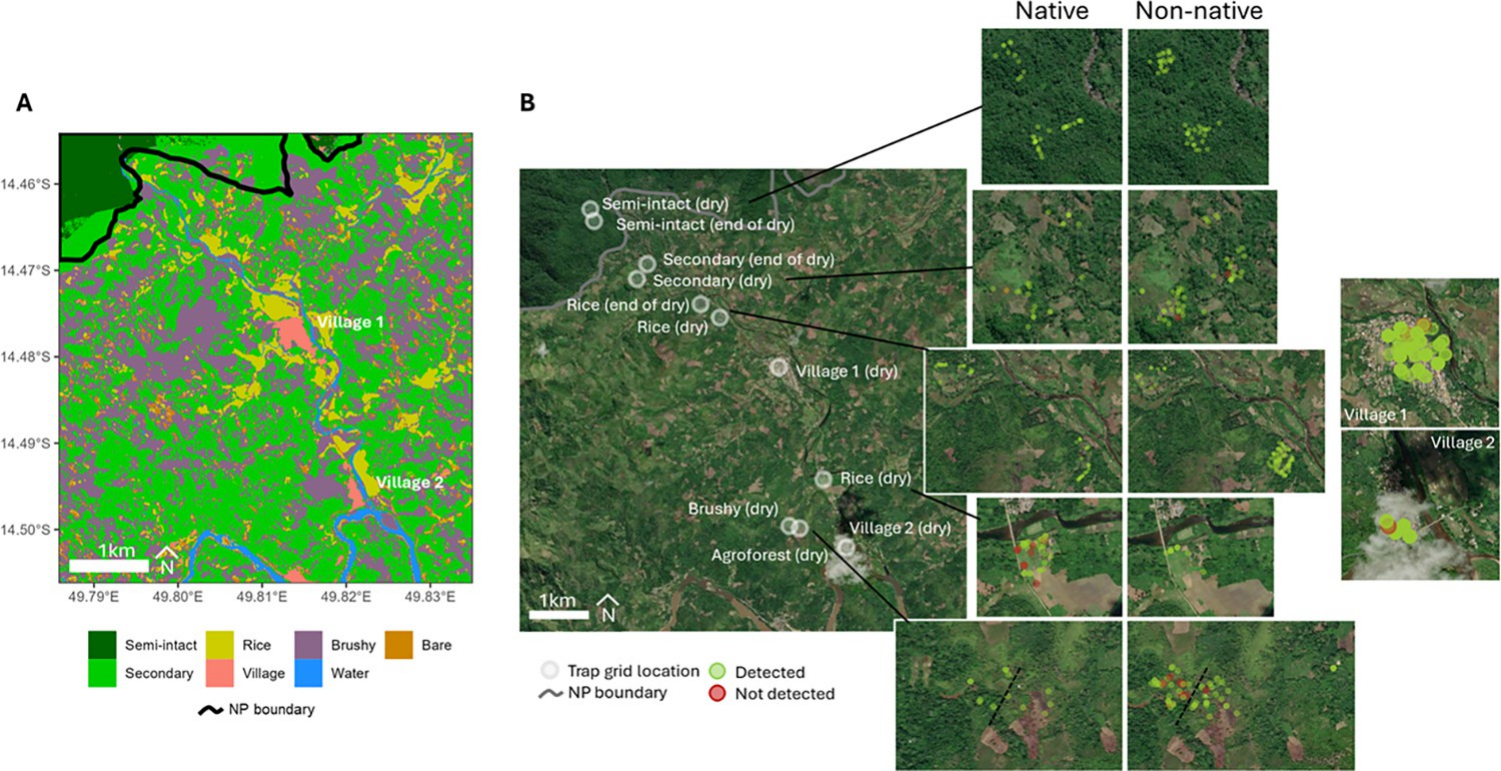
Astrovirus detection in small terrestrial Malagasy mammals captured in the different habitat types in and around the Marojejy National Park. (A) Map presenting the different habitat types at the study sites and (B) map showing the locations of each trapping grid together with the exact position of trapped animals colored in green (AstV negative) or red (AstV positive). The map was obtained using leaflet R package and the Map image is the intellectual property of Esri and is used herein under license. Source: Esri, i-cubed, USDA, USGS, AEX, Getmapping, Aerogrid, IGN, IGP, UPR-EGP, and the GIS User Community (https://arcg.is/1uyPfH).

This study was carried out in northeastern Madagascar, in the SAVA Region, within and adjacent to Marojejy National Park, in the Manantenina River valley and based at a campsite (-14.4662, 49.7986), 2 km from the village of Mandena. This region of the island is well-known for the cultivation of vanilla. Forested areas that have been transformed into a form of agroforestry are composed of largely non-native tree plantations or fields suitable for vanilla and rice cultivation. These modified habitats still hold some endemic species of small mammals [17,19]. Small mammals were trapped across six habitat types encompassing a range of forest cover and human use (See Fig 1). Forest habitat types included semi-intact forest and secondary forest of the lowland moist evergreen forest formation, as defined by Gautier et al. (2018) [20]. The other non-forested habitat types were crop fields and included mixed agriculture, paddy rice, vanilla, and *savoka* (regenerating secondary vegetation after forest clearing, and generally composed of non-native and invasive plants). In many cases, the initial incentive for forest clearing was to establish areas of shifting or swidden agriculture (*tavy* in Malagasy). This type of agriculture depletes the soil of nutrients, only remains productive for a few seasons then the site is abandoned, and subsequently develops to dense brushy regrowth or *savoka*. Each habitat type was sampled during two different seasons: dry season (June-August 2018, Mandena and June-July, Manantenina) and wet season (Novembre-Decembre 2017, Mandena).

### Animal capture and handling

Small mammals were trapped as previously described [17] and included introduced Muridae rodents and Soricidae shrews, as well as endemic rodents (subfamily Nesomyinae) and tenrecs (family Tenrecidae). All animals were identified in the field based on a range of external characters [21] and for some collected individuals based on cranio-dental features. The study included 515 animal samples, of which 375 were trapped in 2017/2018 and were previously screened for pathogenic *Leptospira* [17] while 140 were sampled in 2019 following the same exact trapping methods. Euthanasia was performed by cervical dislocation except for the larger tenrecs (*Setifer setosus* and *Tenrec ecaudatus*) for which a lethal dose of xylazine was administered via inter-muscular injection. For euthanized mammals (all introduced animals, as well as a subset of native animals, typically less than 10 per site), kidney, bladder, lung, colon, urine, blood, and ectoparasites were collected and immediately stored in 75% ethanol. Samples were stored at room temperature in the field and then brought to the laboratory where they were stored at -80°C. All mammal specimens were deposited in the Université d’Antananarivo, Mention Zoologie et Biodiversité Animale, and formerly the Département de Biologie (UADBA). Final species identification of the specimens was made by small mammal specialists based on external and, in some cases, cranio-dental morphological characters.

### Nucleic acids extraction, PCR diagnosis, and sequencing

We used colon tissue samples for the screening of AstroV. Since tissues were stored in EtOH70%, colon samples were rehydrated for 24 hours with osmosis water at 4°C before nucleic acid extraction. Following rehydration for each sample, approximately 20 mg of tissue was placed in 200 μL of ATL buffer (QIAGEN, Valencia, California, USA) containing 20 μL of Proteinase K. These samples were incubated at 56°C for 6 hours to achieve complete lysis and then centrifuged for 15 min at 16 000 rpm. The supernatant was added to 180 μL of VXL buffer (QIAGEN, Valencia, California, USA) and nucleic acid extraction was performed using the Cador Pathogen kit and the QIAcube XT automated system (QIAGEN, Valencia, California, USA). Reverse transcription was performed from 10 μL of nucleic acids using ProtoScript II Reverse Transcriptase and Random Primer 6 (New England BioLabs, Ipswich, Massachusetts, USA) under the following thermal conditions: 70°C for 5 min, 25°C for 10 min, 42°C for 50 min, and 65°C for 20 min, with an Applied Biosystems 2720 thermal cycler (Thermo Fisher Scientific, Waltham, Massachusetts, USA) [14]. Produced cDNAs were tested for the presence of the AstVs RNA-dependent RNA polymerase (RdRp) using a semi-nested PCR [22] with the GoTaq G2 Hot Start Green Master Mix (Promega, Madison, Wisconsin, USA). After electrophoresis on 2.0% agarose gel stained with 2% GelRed (Biotium, Hayward, California, USA), PCR products of the expected size were gel purified with the QIAquick purification kit (QIAGEN, Valencia, California, USA). Sanger sequencing was performed on both strands (Genoscreen, Lille, France) and only those samples for which we obtained an AstV sequence were considered positive in the analyses.

### Statistical analyses

We compared the positivity rates among habitat types and species using Fisher’s exact tests. To investigate which factors predicted AstV infection in animals, we performed model selection based on the corrected Akaike information criterion (AICc) using the dredge function in the MuMIn package in R (package version 1.47.1, Barton 2022). All possible additive models containing any or all of the set of predictors: habitat type (secondary forest, agroforest, brushy regrowth, flooded rice, village), season (end of dry, dry), and the village (Mandena, Manantenina) in which the animal was captured, and the species (*Rattus rattus*, *Mus musculus*), sex (male, female), and mass (g) of the individual. A total of 64 models were considered. We retained all models in which a cumulative sum of Akaike weights less than or equal to 95% to estimate averaged model coefficients and parameter importance (sum of weights across all models in which the variable appears).

### Phylogenetic analysis

All RdRp sequences were included in a phylogenetic analysis with one exception as the sequence was too short (185 bp). A Maximum-Likelihood (ML) analysis was performed with the 21 remaining sequences obtained in the study (GenBank accession numbers ON652986-ON653001 and OP727997-OP728001) and 69 reference AstV RdRp partial nucleotide sequences. The sequence alignment was obtained with Clustal Omega 1.2.3 (with initial and refinement iteration guide trees obtained by fast clustering; mBed algorithm). The ML analysis was performed with the program PhyML 3.1 [23], with the TVM model, an estimation of the proportion of invariable sites (I) and of the nucleotide heterogeneity of substitution rates (α), as selected by Model Generator 0.85 [24], and 1000 bootstraps.

## Results

AstV RdRp was sequenced in 22 of the 515 samples tested (Table 1). Only one individual of the most abundant endemic species living outside of native forest areas tested positive (*Microgale brevicaudata*, family Tenrecidae, n = 98). That animal was captured in secondary forest. The remaining positive samples were from introduced rodents Muridae *Rattus rattus* (5.6%, 15/266) and *Mus musculus* (7.7%, 6/78). Given that these two species had the highest positivity rate, we focused our statistical analyses on them (n = 296). No significant difference in the infection frequency was detected between *Rattus* and *Mus* (Fisher’s exact test, p = 0.59). Positivity rate was significantly higher in Manantenina in 2019 (12.1%, 17/140) than in Mandena in 2017 (0.9%, 1/112) or 2018 (1.5%, 3/259; p<0.001).

**Table 1:** Astrovirus detection in small terrestrial Malagasy mammals captured in and outside of Marojejy National Park. Species that were found positive (*i*.*e*. positive through hemi-nested PCR and with a Sanger sequence) are emphasized in bold. “E” refers to Endemic small mammals and “I” refers to Introduced small mammals.

Species	E / I	Semi-intact forest	Secondary forest	Brushy regrowth	Agroforest	Flooded rice	Village
*Eliurus* *ellermani*	E	NA	0/1(0)	NA	NA	NA	NA
*Eliurus webbi*	E	0/2(0)	0/3(0)	NA	NA	NA	NA
*Nesomys* *audeberti*	E	0/2(0)	NA	NA	NA	NA	NA
Microgalebrevicaudata	E	0/31(0)	1/17(5.88)	0/5(0)	0/3(0)	0/42(0)	NA
*Microgale longicaudata*	E	NA	NA	NA	0/1(0)	NA	NA
*Setifer* *setosus*	E	0/5(0)	0/3(0)	0/2(0)	0/2(0)	0/6(0)	NA
*Tenrec* *ecaudatus*	E	NA	NA	NA	NA	0/2(0)	NA
Rattusrattus	I	0/43(0)	2/43(4.65)	6/23(26.09)	1/14(7.14)	3/81(3.7)	3/62(4.84)
Musmusculus	I	NA	NA	1/9(11.11)	0/6(0)	5/22(22.73)	0/41(0)
*Suncus* *etruscus*	I	NA	0/5(0)	0/1(0)	NA	0/7(0)	NA
*Suncus* *murinus*	I	0/1(0)	0/2(0)	NA	0/2(0)	0/14(0)	0/12(0)

**Fig 2:**
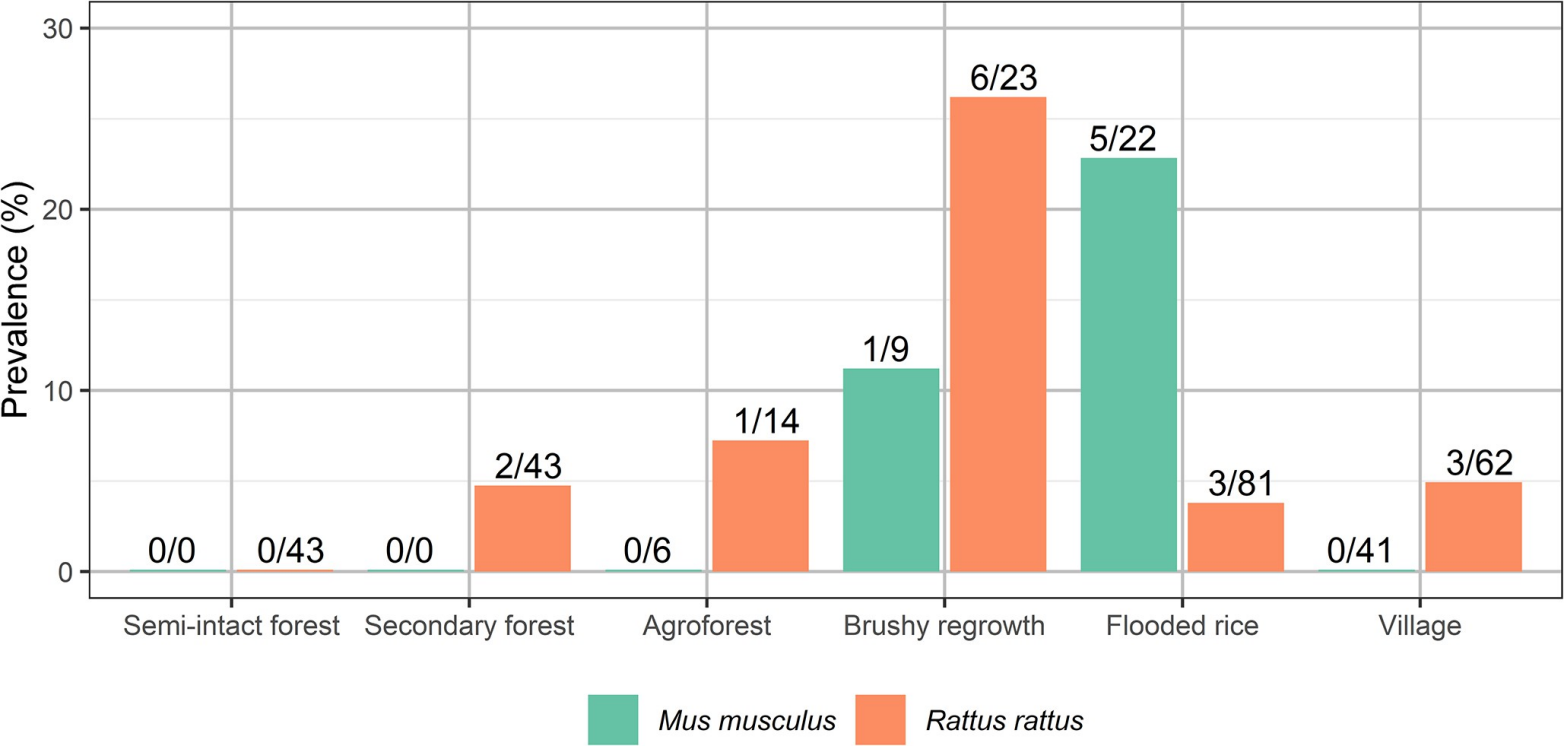
Astrovirus positivity rates in introduced rodents according to habitat, in and outside of Marojejy National Park (Madagascar).

Positivity rates varied by species and habitat (see Fig 2 for introduced rodents). Overall, the highest positivity rate of AstVs infecting small mammals was in brushy regrowth (17.5%, 7/40), and the lowest in flooded rice fields (4.6%, 8/174), secondary forest (4.1%, 3/74), agroforest (3.6%, 1/28), village (2.6%, 3/115), and semi-intact forest (0%, 0/84). These differences in overall positivity rates were statistically significant with a Fisher’s exact test (p = 0.005; Fig 2). The frequency of infected individuals showed a trend to be slightly higher in brushy regrowth than in semi-intact forest (6 vs 0, pairwise Fisher’s tests with Bonferroni correction, p = 0.062) and in brushy regrowth than in village settings (6 vs 3, p = 0.067).

**Fig 3:**
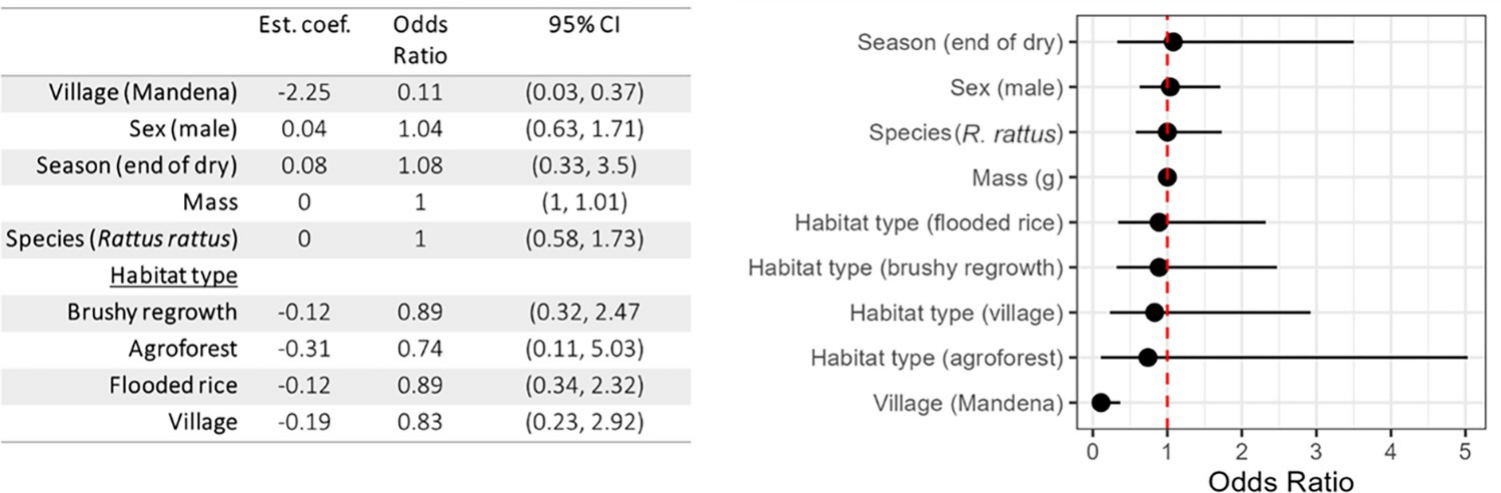
Model-averaged results of logistic regression predicting AstV infection based on habitat type (with semi-intact forest as the reference level), species (with *Rattus* as the reference level), sex (with female as reference level), and body mass.

The 95% cumulative sum Akaike weight subset of models contained 19 models. The highest weighted model (weight = 0.254) had only one parameter: village. The remaining models had ≤0.10 weights. The sum of weights of each predictor as determined by the sum of weights of each predictor in all models in descending order are village (weight = 1.00) across all 19 models, sex (weight = 0.26) across 8 models, season (weight = 0.24) across 7 models, mass (weight = 0.24) across 7 models, species (weight = 0.24) across 7 models, and habitat type (weight = 0.12) across 5 models. Despite their presence in the best model subset, the 95% CI of all of these estimates contained 1, thus none of these predictors significantly impacted the odds of infection, with the notable exception of “village”. Full model-averaged parameter estimates are provided in Fig 3. The complete table of all model combinations and coefficients is provided in S1 Table.

A second model with only the data from the village of Manantenina was considered because positivity rate was significantly higher there (n = 102). The full model investigated the effect of four variables: habitat type (secondary forest, agroforest, flooded rice, village), species (*Rattus rattus*, *Mus musculus*), sex, and mass. Season was not considered because trapping only occurred in the dry season. Across the 16 models, the intercept only model had the highest Akaike weight (0.21), and the remaining models had ≤0.16 weights. The sum of weights of each predictor in descending order were habitat type (weight = 0.37) across 5 models, sex (weight = 0.32) across 5 models, mass (weight = 0.25) across 5 models, and species (weight = 0.22) across 4 models. Full model-averaged parameter estimates are provided in S2 Table.

**Fig 4:**
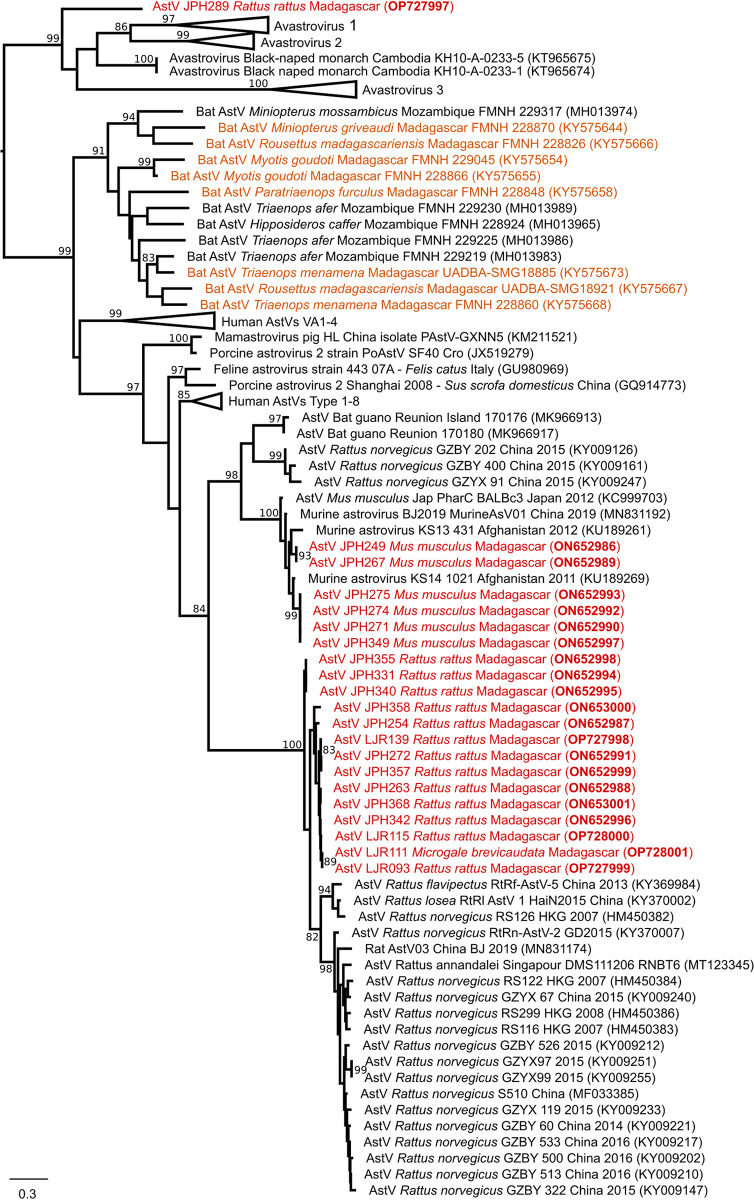
Midpoint rooting maximum likelihood tree obtained with 90 Astrovirus (AstV) RNA-dependent RNA-polymerase partial nucleotide sequences (389 bp). The phylogenetic analysis was conducted with the TVM + I + Г evolutionary model (I = 0.14; α = 1.04) and 1000 bootstraps. Sequences derived from this study are colored in red. AstVs previously detected in Malagasy bats are colored in orange. Bootstrap values are reported when higher than 80. Scale bar: mean number of nucleotide substitutions per site.

We conducted a phylogenetic analysis of the AstVs that were detected in these small mammals. The overall sequence pairwise similarity among the 21 obtained sequences ranged from 46% to 100% and clustered in three phylogenetic groups (Fig 4). The first group included sequences obtained from AstVs found in *Rattus rattus* (n = 14) and the one positive *Microgale brevicaudata*. The second group contained only sequences from *Mus musculus* (n = 6), clustering with an AstV sequence reported from Afghanistan. The remaining AstV sample detected in *Rattus rattus* clustered at the root of the avian astrovirus clade (i.e., the Avastrovirus clade). Of note, none of the sequences obtained in this study were related to those obtained from AstVs detected in Malagasy bats (Fig 4).

## Discussion

Although there is no information regarding the exposure of the Malagasy population to AstVs, presented data suggest that human populations they may be exposed to those viruses, at least in rural settings. The screening of 515 small terrestrial mammals revealed that Astrovirus infection is almost exclusively limited to introduced rodents, which typically are more common in habitats disturbed by human activities, as compared to native forest formations [25]. We found only a single individual of an endemic species infected with AstVs, *Microgale brevicaudata*, which is one of the few endemic species able to adapt to human disturbed habitats in this area of Madagascar [17,26]. However, the relatively small number of samples of endemic Nesomyinae rodents and Tenrecidae tenrecs limits our ability to assess the importance of these mammals in AstV maintenance at the study site. It is also noteworthy that introduced rodents exhibited AstV infection but not introduced shrews (genus *Suncus*, Table 1), in contrast with a recent investigation reporting 7.4% of AstVs infection in *Suncus murinus* in Singapore [9].

The phylogenetic tree of the AstVs is structured by host species, with one strongly supported clade composed of sequences from mice, a second supported clade composed of viral sequences obtained from rats with the notable exception of an AstV sequence from endemic *Microgale brevicaudata*, and a last sequence obtained from a rat and clustering in the Avastrovirus clade, which typically infects birds. Of note, no birds were sampled at the study site and the obtained sequence was distinct from any sequence available in databases, so the possibility of a contamination must be considered as low. The topology of the phylogenetic tree and infection almost exclusively limited to introduced rodents support that these AstVs were introduced to Madagascar with murid rodents and with limited spillover to endemic species. Current archaeological evidence indicates that *Mus* was introduced to the island during the 11th century via Islamic trade routes [27] while the introduction of *Rattus rattus* on Madagascar is thought to have originated from India or the Middle East [28,29]. Further investigation on the origin of rodent AstVs in Madagascar could be assessed by sampling additional rodents in Madagascar and in the Middle East, along with more detailed studies of virus circulation in the islands of the western Indian Ocean and neighboring East Africa. Major differences between rodent and bat AstVs also suggest that these viruses arrived on Madagascar by different dispersal events, with the bat viruses via migration from continental areas and the rodent viruses via human introductions.

On Madagascar, *Rattus rattus* is highly adaptable, found in diverse habitats across the sampled gradient from semi-intact forest to secondary growth and agricultural fields, and in village homes [30]. It is the most frequently captured rodent in many field surveys on the island, including those in forests transformed by human activities [31]. Although introduced rodents were present in all sampled habitats, some results indicated that habitat type influences positivity rate, with the lowest positivity rate found in semi-intact or secondary forests. This pattern may result from small mammal community structure sheltered by these less disturbed habitats: introduced rodents exist at lower abundance in our sampled forest habitats, which could lower transmission among competent reservoirs. The majority of cases were found in the brushy regrowth (n = 6) and flooded rice fields (n = 8), with the latter sheltering an abundant *Mus* population displaying the highest positivity rate in our study. Despite seeing significant differences between species and habitat types using pairwise comparisons we were unable to determine what demographic/morphometric indices (species, age, sex, mass) might be driving these differences using a GLM. However, given the number of covariates in the models and the low positivity rate, only a strong effect would have been significant in our models. Several studies have highlighted the ability of AstVs to remain infectious for several weeks in surface water and especially in groundwater due to favorable temperature, limited exposure to bacterial agents [32–35]. Such a high persistence ability may favor transmission among *Mus* of AstVs in rice fields. However, this pattern was not observed in *Rattus*, and hence deserves cautious interpretation. Furthermore, we found that the village in which animals were trapped was the most important predictor of AstV infections, with positivity rate being substantially higher in Manantenina (12.1%, 17/140) than Mandena (1.3%, 5/375, p<0.001 fisher’s exact test). In Manantenina, habitat type was a weak predictor of infection. Because sampling was conducted asynchronously between villages, we are unable to determine if this effect is due to a true difference between the villages or varying annual positivity rate of AstVs.

The detection of a “*Rattus*” AstV in *Microgale brevicaudata* and of an avian AstV in *Rattus rattus* highlights the potential for host shifting of these viruses [36]. This study and a previous investigation on bat AstV [14] provides an initial assessment of the diversity of AstV associated with Malagasy mammals, but additional research is needed to investigate AstVs infection in wild avian hosts, as well as in avian and mammalian livestock and humans, to fully assess AstV spillover in natural and human-modified habitats.

Our study has some obvious limitations, including the randomness of the sampling strategy. Although the trapping design aimed at trapping most of the small mammal diversity, some species might have been under sampled, especially endemics. Our study thus does not allow addressing the importance of endemic rodents in the maintenance of AstVs because of the low number of sampled animals and because these endemic rodents might shelter AstV that are not detected by our screening PCR scheme. However, these species have few contacts with humans as they mostly occupying intact or semi intact forests. Altogether, presented data have implications for the potential transmission of zoonotic pathogens from introduced rodents to people, especially in degraded habitats, although to our knowledge there is no available data on AstV epidemiology in human populations from Indian Ocean islands. Firstly, the topology of the presented phylogenetic tree strongly suggests that AstVs infecting introduced rodents at the study site were introduced to Madagascar, likely from Asia, although complementary studies such as the investigation of AstVs on the other different islands of the region are necessary to confirm this. Secondly, our findings show that human exposure increases in disturbed habitats due to (i) a higher AstV positivity rate in animal reservoirs within these habitats, as well as (ii) an increased abundance of invasive rodent hosts. The land-use types in which people spend the most time may present the greatest risk of exposure to AstVs from terrestrial small mammals, which in turn has implications for diarrheal diseases in rice farmers. Altogether, our data encourage the need for assessing the actual exposure of the local population, especially farmers, to the AstVs described herein.

## Supporting information

S1 Table Model combinations and coefficients. (XLSX)

S2 Table Full model-averaged parameter estimates. (CSV)
